# An Image Processing Approach to Quality Control of Drop-on-Demand Electrohydrodynamic (EHD) Printing

**DOI:** 10.3390/mi15111376

**Published:** 2024-11-14

**Authors:** Yahya Tawhari, Charchit Shukla, Juan Ren

**Affiliations:** 1 Mechanical Engineering Department, Iowa State University, Ames, IA 50011, USA; cshukla@iastate.edu; 2 Department of Mechanical Engineering, College of Engineering and Computer Sciences, Jazan University, Jazan 45142, Saudi Arabia

**Keywords:** EHD drop-on-demand, feature extraction, edge detection, contour detection, dot size quantification, dot distance quantification

## Abstract

Droplet quality in drop-on-demand (DoD) Electrohydrodynamic (EHD) inkjet printing plays a crucial role in influencing the overall performance and manufacturing quality of the operation. The current approach to droplet printing analysis involves manually outlining/labeling the printed dots on the substrate under a microscope and then using microscope software to estimate the dot sizes by assuming the dots have a standard circular shape. Therefore, it is prone to errors. Moreover, the dot spacing information is missing, which is also important for EHD DoD printing processes, such as manufacturing micro-arrays. In order to address these issues, the paper explores the application of feature extraction methods aimed at identifying characteristics of the printed droplets to enhance the detection, evaluation, and delineation of significant structures and edges in printed images. The proposed method involves three main stages: (1) image pre-processing, where edge detection techniques such as Canny filtering are applied for printed dot boundary detection; (2) contour detection, which is used to accurately quantify the dot sizes (such as dot perimeter and area); and (3) centroid detection and distance calculation, where the spacing between neighboring dots is quantified as the Euclidean distance of the dot geometric centers. These stages collectively improve the precision and efficiency of EHD DoD printing analysis in terms of dot size and spacing. Edge and contour detection strategies are implemented to minimize edge discrepancies and accurately delineate droplet perimeters for quality analysis, enhancing measurement precision. The proposed image processing approach was first tested using simulated EHD printed droplet arrays with specified dot sizes and spacing, and the achieved quantification accuracy was over 98% in analyzing dot size and spacing, highlighting the high precision of the proposed approach. This approach was further demonstrated through dot analysis of experimentally EHD-printed droplets, showing its superiority over conventional microscope-based measurements.

## 1. Introduction

Electrohydrodynamic (EHD)-inkjet printing is a non-contact, additive printing technique that uses electrical force to drive the printing liquid [1,2]. The ink is expanded and injected as tiny drops onto the printing substrate for the drop-on-demand (DoD) printing process [3,4,5]. The benefits of this process range from flexibility and functionality to lower downtimes and mass personalization. EHD DoD is one of the promising AM techniques with excellent resolution [4,6,7] since the produced ink droplets are smaller, making it appropriate to fabricate micro/nanoscale designs such as solar cells, micro-array sensors, and micro-LED displays [8,9,10].

The precision of dot profiles in EHD printing is substantial, significantly impacting the quality and functionality of manufactured products [11]. In EHD printing, a high voltage is used to create a jet of liquid that can deposit high-resolution materials [5]. The size of the dot, which is the basic unit of material deposited onto the substrate, is crucial because it directly affects the final product’s resolution and smoothness. Smaller droplets enable higher resolution, producing micro- and nanoscale structures with finer details, such as microarray sensors [12]. Moreover, the consistency in droplet size across the printing process ensures uniformity, which is crucial for applications like micro-LED displays where any variation can affect the overall efficiency and performance [13]. However, the spacing between the dots in DoD EHD printing must be carefully managed to optimize the quality of the final product. Insufficient spacing may lead to overlapping or gaps, compromising the overall structural integrity and disrupting the mechanical and/or electrical connectivity when used in circuitry applications [10,14].

Current approaches to dot analysis in DoD EHD printing mostly depend on manual inspection and estimation utilizing microscope software, a procedure fraught with limitations and difficulties [15]. These methods involve manually identifying and tracing the boundaries of the printed dots on the substrate using a microscope. The analysis assumes that these dots are completely round in order to estimate their sizes, such as diameter and area. These assumptions provide a large potential for inaccuracy because actual droplets in printing can differ from perfect circular forms due to many factors, such as the qualities of the surface they land on, the way the fluid moves, or the environmental temperature. In addition, these approaches frequently exclude essential information regarding the spacing between dots, which is necessary to guarantee precise DoD printing functionality. The lack of accurate and automated measurement tools in these conventional processes obstructs the capability to replicate prints of superior quality consistently and restricts the thorough examination needed to advance printing techniques, especially in applications that demand precise and uniform results, such as the production in micro/nanoscale. For example, quantifying the size of micro-LED dots for DoD EHD printing faces significant limitations using these techniques, which include low resolution, which restricts the ability to detect minute size variations, poor dot shape, and uniformity assessment, which are critical for achieving consistent brightness and color across the display, as well as challenges in accurately quantifying overlay registration [16,17,18]. These quantification challenges can compromise the final product’s overall performance and aesthetic quality.

Furthermore, the manual process of current dot analysis approaches leads to extra errors due to human error and subjective interpretations regarding the estimation of a dot’s boundary. The manual approach is also time-consuming, restricting the production throughput in DoD EHD manufacturing. Therefore, there is a vital requirement for advanced and automated image processing methods that can precisely detect and analyze the printed droplets without human operators’ subjective biases and limits. This demand is vital for applications that require high-resolution and flawless printing, hence expanding the capability of EHD inkjet technology. In recent years, recognition applications in the image processing domain have become essential in multiple fields, performing as practical tools for automated monitoring, automated methods for monitoring and quality control, and real-time decision-making. In civil engineering applications, Structural Health Monitoring (SHM) systems, with the help of vision computation, can detect various structural events or phenomena, such as cracks or deformations, on bridges and other vital structures to enhance safety and maintenance efficiency [19]. Similarly, in agriculture fields, lightweight object detection models with restricted modifications in YOLO (You Only Look Once) are implemented to detect and classify crops like pitaya under different lighting conditions to optimize and enhance yield estimation and harvesting processes [20]. In the medical field, recognition techniques help detect tumors, abnormal tissue, and other health signs to improve the diagnosis period and the treatment strategies [21]. Robotics also incorporates object recognition algorithms used to navigate or manage robotic tasks in different complex environments, such as warehouses or disaster response situations, which enhance operations and safety [22]. These recognition advancements support industries that emphasize the need for development in automated image analysis in high-precision applications concerning the quality needed in DoD EHD manufacturing and other specialized domains.

Advances in computer vision techniques, such as edge detection, pattern recognition object detection, and image segmentation, have been broadly used in image processing applications and can potentially address the issues mentioned above [23,24,25,26]. Therefore, this work aims to develop an automatic quality analysis approach for DoD EHD printing. Considering the fact that the EHD DoD printed dots do not have fixed shapes (e.g., circles or ellipses), dot recognition in the proposed method does not rely on techniques that are designed for fixed shape recognition, such as Fast Radial Symmetry Transform [27,28] and Hough transform [29]. Specifically, we propose a feature-extraction technique that combines edge and contour detection in image processing to address the challenges associated with dot measurement in DoD EHD printing. Edge detection serves to identify intensity and texture discontinuities that distinguish droplet boundaries from the background [30,31]. At the same time, contour detection traces the entire closed shape of droplet outer boundaries. By employing these techniques, it is possible to enhance the analysis and detection of significant structural boundaries of individual EHD droplets and visualize them within printed patterns. Using edge and contour representations enables accurate measurement of essential features such as droplet sizes, morphology, spacing, and overall pattern configuration. Developing integrated methods for detecting edges and contours helps overcome resolution and precision limitations. Therefore, this work focuses on developing methods to improve the sensing accuracy. The accuracy of the EHD printing droplet is enabled by using the edge detection algorithm to enhance edges and the contour algorithm to detect outlines to determine the shape and position of the droplets in the image and to analyze the printed droplet during operations.

The proposed image processing method consists of three key steps: image pre-processing with Canny edge detection, boundary detection and localization, and dot distance calculation and categorization. In the pre-processing stage, images of DoD EHD printing are first filtered for noise reduction and then processed with a Canny edge detector to find the edges of the printed dots. Then, the detected edges are used as the input for boundary detection to identify the centroid of each dot for dot size calculation. Finally, dot spacings are calculated based on the distance of the identified centroids and categorized using pre-chosen distance thresholds. This approach is entirely software-based and thus does not involve manual handling. The proposed method was first tested using simulated EHD printed droplet arrays with specified dot sizes and spacing, and the achieved quantification accuracy was over 98% in analyzing dot size and spacing, highlighting the high precision of the proposed approach. This approach was further demonstrated through dot analysis of experimentally EHD-printed droplets, showing its superiority over conventional microscope-based measurements.

The paper is structured as follows: The methodology is explained in Section 2. Section 3 applies to the results of experimentation and discussion. The validation is presented in Section 5, while the conclusion is provided in Section 6.

## 2. Methodology

**Figure 1:**
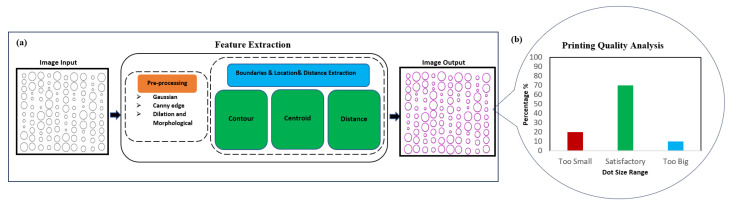
Overview of the proposed framework for DoD EHD printing analysis. (**a**) The proposed method takes a microscope image of printed droplets as the input, outputs the detection results, and calculates the spacing of the dot size (area). (**b**) Visual analysis of the printing quality provides size distribution of the printed dots. The x-axis represents the three categories of dot size range, and the y-axis indicates the percentage within each size category.

This study utilized feature extraction to identify and analyze microscale shapes of DoD EHD printing, as well as to compute distance by integrating OpenCV packages with Python 3.10 and executing the code with the PyCharm command prompt. The framework is illustrated in Figure 1.

### 2.1. Pre-Processing with Canny Edge Detection

**Figure 2:**
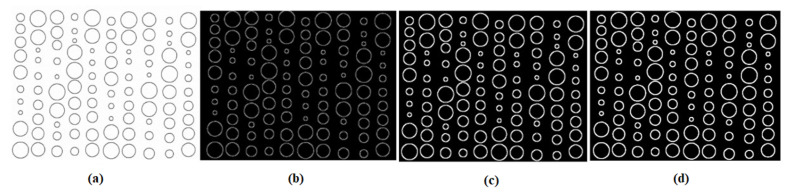
An image (**a**) is used as input for the pre-proposed method; (**b**) is the image after applying a Gaussian filter; (**c**) is the image after applying Canny edge detection; (**d**) is the image after applying dilation and morphological closing.

The initial phase is to enhance the image’s features and reduce noise in order to facilitate the detection and analysis of the printed dots within the image, as illustrated in Figure 2. First, the image contrast and brightness were adjusted through GUI trackbars [32,33,34] based on user requirements, followed by the pre-processing procedures below.

**Assumptions of Proposed Detection Techniques:** The proposed detection techniques assume that the printing environment is controlled and that image quality is sufficient for accurate edge and contour detection. In particular, it is assumed that the images used in the analysis have low noise levels and good lighting conditions to get better canny edge detection and contour tracing results. Furthermore, the technique assumes that the printed droplets exhibit consistent contrast against the substrate, which edge detection algorithm can accurately differentiate between the edges of the dots and the background.

**Noise Reduction via Image Filtering:** This step implements an image filter, such as a Gaussian filter, in order to achieve image smoothing, which is essential for minimizing noise and eliminating irrelevant details from the input. A Gaussian filter is chosen as the Gaussian noise, which is uniformly spread across the image and better reflects the actual noise usually encountered in EHD printing images. Gaussian filtering provides effective noise suppression while maintaining the integrity of the dot edges. The following Gaussian filter formula is used to produce an output image with less noise [35].

(1) $$G\left(x,y\right)=\frac{1}{2\pi \sigma^{2}}\exp\left(-\frac{x^{2}+y^{2}}{2\sigma^{2}}\right),$$

where *x* and *y* represent the distances from the origin along the horizontal and vertical axes, respectively, and σ represents the standard deviation of the Gaussian distribution.

**Edge Detection for Identifying Object Edges:** Following image smoothing, the edge detection approach is applied to identify the edges of the printed dots precisely. This step is crucial in the overall edge detection process and is achieved using the Canny Edge Detection method [36,37,38], which consists of multiple steps, including the following:

- Apply the Sobel operator [39] to calculate the gradient’s magnitude (*G*) and direction (θ) at every pixel.
  (2) $$G=\sqrt{G_{x}^{2}+G_{y}^{2}},$$
  (3) $$\theta =\tan^{-1}\left(\frac{G_{y}}{G_{x}}\right),$$
  where the gradients in the horizontal and vertical directions, denoted as $G_{x}$ and $G_{y}$, respectively, are calculated by the Sobel operator.
- Use Non-maximum Suppression [40] to narrow edge widths to a single pixel, retaining only those pixels at the peak of the gradient magnitude.
- Apply Hysteresis thresholding to distinguish strong, weak, and non-edge pixels. This step ensures that only strong and weak pixels connected to well-defined edges are identified, i.e., true edges.

The threshold settings directly determine the edge detection sensitivity, influencing the algorithm’s ability to distinguish true and false edges. They can be determined using a trial-and-error method or histogram analysis. For this step, the input is the blurred image. After processing, the output will have sharper and more connected edges. This is achieved by applying non-maximum suppression and hysteresis thresholding techniques, as shown in Figure 2c.

**Enhancement of Detected Features Through Morphological Dilation and Closing:** Dilation and closure are two morphological processes employed for image enhancement [41], as depicted in Figure 2d. Dilation is used to increase the size of the edges by adding pixels, which enhances the visibility of the image’s features. Subsequently, the closing method applies morphological closure to connect small gaps and fill in holes in the detected edges, leading to a more cohesive representation of the image’s features.

Algorithm 1 provides a clear plan for executing pre-processing procedures iteratively. image by image. The final output of the pre-processing is an image with patterns with well-defined edges and reduced noise, setting the foundation for detailed analysis next.
**Algorithm 1:** Pre-possessing **procedure** Analysis(image, feature extraction)       importOpenCvLibraries       GUI trackars for Canny thresholds       Createplaceholderwindowsforimage       Createatrackbartotuneimageedges         Create Pre-Processing Function       **Define Pre-Processing function**             Noisereduction             Edgedetection             Dilation             Morphological closing       **return closed**       **while** True **do**             **if** *webcam is capturing video from cameras* **then**                   **load video**             **else if** *webcam is not capturing video* **then**                   **load image from file**                   *Pre-Processing (image)*

### 2.2. Boundaries Extraction and Localization

This phase executes dot analysis and localization by utilizing contour detection and moment calculation. The principal aim of this enhanced methodology is to identify, analyze, and approximate the centroid of dot shapes by employing their boundaries with significant measure features. To achieve accurate identification and analysis of dot features, the contour detection procedures (as shown in Algorithm 2) contain a combination of the following steps.
**Algorithm 2:** Boundaries and Localization**Apply Contours in the Image Preprocessed**Find Contours**Loop Over to Analyze Each Contour****for** *each detected contour* **do do**       Contour area       Draw contour       Contour perimeter       Contour approximation       **Calculate Centroid**       Apply moments function to find shapes center       append shapes center coordinates

**Dot identification through finding contour points:** This step is to identify regions of shapes or boundaries in the image improved from pre-processing. The “Find Contours” function is used to extract the edges of dots within an image [42]. It involves extracting the boundaries of the dot and saving them as an array that contains the coordinates of the vertices. This enables the rendering of contours and the quantification of the dimensions of each individual dot. By selecting the retrieval mode, the algorithm emphasizes the detection of the most obvious external outlines. This is crucial for distinguishing individual dots without considering their internal features.

Additionally, a contour approximation method is used to accurately capture every point that lies on the boundary of the contour. This is essential for identifying all boundaries in order to subsequently calculate the area of each dot. The contours are stored as a Python list of NumPy arrays containing (*x*, *y*) coordinates that collectively define the entire contour. This setup facilitates the process of identifying and preparing dots within the image for further actions.

**Dot area analysis through stored contour points:** Analysis is then performed for each detected contour. Specifically, a contour area function is used to calculate the area of each dot, as indicated by its vertex coordinates, which are commonly located along the boundaries of the dots. The dot area is calculated using the following Shoelace formula, which entails summing the cross-products of consecutive vertex coordinates and dividing the result by half [43].

(4) $$A=\frac{1}{2}\left|\sum_{i=1}^{n}\left(x_{i}y_{i+1}-x_{i+1}y_{i}\right)\right|,$$

where *A* represents the area of the contour. The points $(x_{i}\;y_{i}$) and $(x_{i+1}\;y_{i+1}$) are consecutive vertices of the polygon, and the summation is performed over all vertices of the polygon. The absolute value is taken to ensure the calculated area is positive. This method is particularly advantageous when it comes to precisely quantifying the areas of dots that possess asymmetrical shapes.

Moreover, the “Draw Contour” function is applied to trace and outline the dots (by connecting the counter vertices using eye-catching colors, as shown in Image Output in Figure 1). This add-on feature is to enhance the demonstration of the proposed method visually.

**Dot centroid through estimation of its center:** Once the dot area is determined, the next is to estimate the shape of the dot identified in an image and calculate the centroid. This stage first uses the contour perimeter function to determine the perimeter of a contour identified. Contour approximation is then applied for shape approximation of the contour, resulting in a simpler dot shape with fewer vertices. An approximation parameter, essentially a fraction of the contour’s perimeter, is used to determine how closely the approximated shape should adhere to the contour [44].

Next, the centroids of the validated shapes are computed using the moments function. The moment’s function is a statistical measurement that outputs the center, e.g., the geometric center, of any validated shapes by calculating the coordinates of the center ($c_{x}$, $c_{y}$), as follows:

(5) $$c_{x}=\frac{M_{10}}{M_{00}},$$

(6) $$c_{y}=\frac{M_{01}}{M_{00}},$$

where $M_{10}$, $M_{01}$, and $M_{00}$ are the spatial moments of the shape.

### 2.3. Distance Estimation and Categorization

The quality of DoD EHD printing is closely related to the spacing of the printed dot. The calculated centroids of the validated dots can be used for quantifying the dot spacing and quality classification. Before proceeding with the printing quality analysis, the centroids are sorted based on their coordinates and then stored in a list. The sorting process makes sure the distances calculated are those of neighboring dots. The algorithm (see Algorithm 3) then iterates through the sorted centroids, computes distances between them, and outputs a table containing the distances between each pair of calculated distances.
**Algorithm 3:** Distance and CategorizationThe order of the shapes center on the listSort shapes center to list**for** (*i*, *center*) in sorted centers **do**      Print(*i* + 1, *center*)Calculate Distance Between Centroids**for** iinrange(lengthofsortedcenters−1) **do**      $C_{1}$ = (*sorted centers*[i+1][0] − *sorted centers*[*i*][0])      $C_{2}$ = (*sorted centers*[i+1][1] − *sorted centers*[*i*][1])      $distance=\sqrt{(}C_{1}^{2}-C_{2}^{2}$)      Distance Categorization      **if** distance<100 μm **then**           Classify as close distance      **else if** distance>200 μm **then**           Classify as far distance    **else**           Classify as satisfied distanceStack and display processed images in each stageApply image stacked functionUsed image display function

**Dot distance calculation:** The primary analytical part of this step computes the Euclidean distance between consecutive centroids from the sorted list. The calculation is executed within a loop that iterates through the sorted centroids, utilizing the mathematical formula for Euclidean distance, i.e.,

(7) $$d_{ij}=\sqrt{{\left(c_{xi}-c_{xj}\right)}^{2}+{\left(c_{yi}-c_{yj}\right)}^{2}}.$$

**Dot Distance categorization:** The calculated distances are categorized into three distinct groups: close, far, and satisfactory. These categories are determined by user-selected thresholds. If the distance is less than the chosen threshold’s lower bound, it is considered to be close. If the distance remains within the threshold range, it is considered satisfactory. If the distance exceeds the upper bound of the threshold, it is considered far. These categories provide a simple yet effective way to present the printing quality and can help in the printing parameter optimization. For example, too many “Close”/“Far” distances indicate slow/fast lateral stage movements or high/low jetting frequencies during the printing, respectively.

**Displaying the quantification results:** Besides the aforementioned dot analysis, the proposed algorithm also displays the results for visual inspection and interpretation, which include both the identified shape contour and centroid for each dot on the same image.

## 3. Experiments

**Figure 3:**
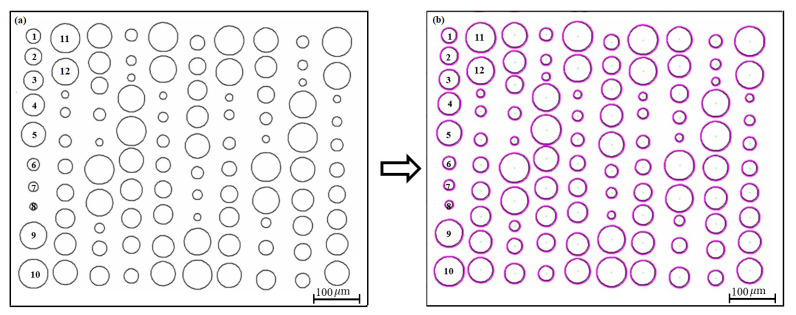
Line dot printing pattern. (**a**) original pattern. (**b**) processed pattern.

The proposed algorithm was initially validated using artificially generated test images, which contained dots with known sizes (areas) and spacing information. SolidWorks was used to create these images (for two different printing patterns) of varied dot sizes and spacings. The first test image (as shown in Figure 3), mimicking a line-by-line printing pattern, contained 100 dots with different sizes and distances. Another test image used a circular printing pattern, which had 12 dots, all with different sizes. The centroids of neighboring dots along the printing direction were selected to determine the dot distances. Moreover, for demonstration, the proposed approach was compared with the broadly used microscope-based manual quantification method for analyzing experimental EHD DoD printing results in terms of speed and accuracy.

In the experiment, a GaussianBlur filter with a kernel size of 7 × 7 was applied to the images for optimal performance. We also used GUI trackers with pixel gradients ranging from 220 to 255 for upper and lower thresholds. Note that the Canny threshold settings can be determined using a trial-and-error method or histogram analysis. For dot distance analysis, the distance threshold values were chosen as if the distance is less than 100 μm, it is classified as “close”; if the distance is greater than 200 μm, it is classified as “far”; and for distances between 100 μm and 200 μm, the classification is “satisfactory”. These distance values were determined based on the desired printing requirement.

## 4. Results

### Image Analysis Algorithm Validation

**Validation for line pattern test image:**Figure 3 shows the first test image, which contains 100 circular dots with different sizes (radii ranging from 15 to 60 μm) and distances, which mimics the lines printed by EHD DoD printing. Each column represents a printed line, with sequential numbering from top to bottom. Figure 3a presents the original image pattern, while Figure 3b displays the image processed by the proposed analysis algorithm. Once feature extraction was applied, the dots were identified and annotated, along with their boundaries. This annotation helps in visualizing and verifying the accuracy of the proposed approach and provides clear comparison information between the true and estimated dot areas. Sizes of the dots were quantified as the dot areas, and the dot distances were estimated as that of the centroids of neighboring dots along the printing direction (top–down direction).

The proposed approach was able to quantify the dot sizes accurately. The overall quantification accuracy for all 100 dots was 92.5 ± 3.9%. For example, Table 1 presents data on generated dots with various radii ranging from 15 to 60 μm. It lists the true area (i.e., $\pi \times {\mathrm{radius}}^{2}$) and the calculated values using the proposed approach, along with the calculation accuracy with respect to the true values. For instance, a circle with a radius of 35 μm has a true area of 3848.45 $\mu m^{2}$, and the estimated area of 3846.09 $\mu m^{2}$ yields an accuracy rate of 99.94%. For most of the dot sizes, the image processing algorithm yielded similar area estimation accuracy. However, the area quantification error became notably larger for smaller dots, such as the one with a radius of 20 μm. This discrepancy is primarily due to the pixel-based detection mechanism of the algorithm, where the pixel-to-feature size ratio significantly impacts the accuracy of smaller features. In such cases, even a single miscounted pixel can lead to a notable percentage estimation error. This can be easily avoided by capturing images with high-resolution cameras.

**Figure 4:**
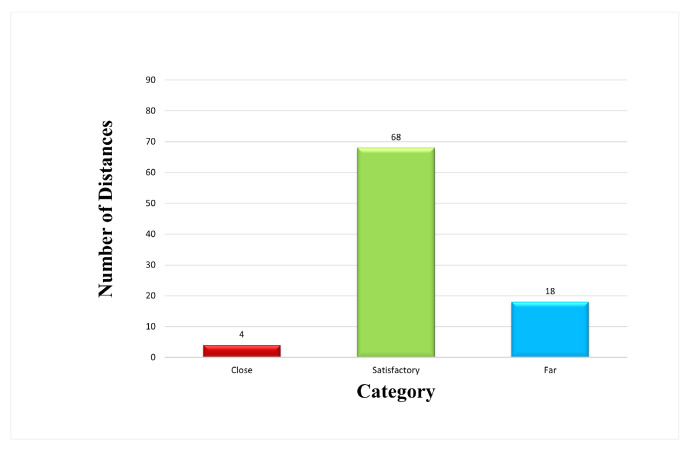
Distance Quantification Analysis.

The distance of the neighboring dots in each line (i.e., column) was quantified for analyzing dot space as the assumed printing direction was vertical. The overall distance quantification accuracy was high, 98 ± 1.6%. For example, Table 2 shows the distance information of the dots labeled in Figure 3. The algorithm also classified the distance based on pre-chosen criteria: dot spacing in the range of 100 to 200 μm was considered satisfactory, for example. Furthermore, Figure 4 illustrates the dot distance analysis results, which reflect the overall printing quality. Of the ninety distances quantified (ten lines and ten dots in each line), sixty-eight of them satisfied the chosen criteria, four of them were too close, and eighteen of them were too far. This information is important in optimizing the EHD printing parameters, such as jetting frequency and stage moving speed.

**Figure 5:**
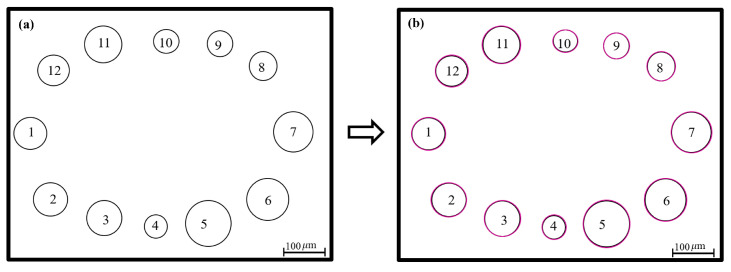
Circular dot printing pattern. (**a**) original pattern. (**b**) processed pattern.

**Validation for circular pattern test image:** Figure 5 shows the second test image, with 12 dots, which mimics the circular pattern of EHD DoD printing (counterclockwise direction). Figure 5a presents the original image pattern, while Figure 5b displays the image processed by the proposed analysis algorithm. Sizes of the dots were quantified as the dot areas, and the dot distances were estimated as that of the centroids of neighboring dots along the printing direction (counterclockwise direction).

The proposed approach was able to quantify the dot sizes accurately. The overall quantification accuracy for all 12 dots was 98.8 ± 0.7%. For example, Table 3 presents data on generated dots with various radii ranging from 6 to 12 μm. It lists the true area (i.e., $\pi \times {\mathrm{radius}}^{2}$) and the calculated values using the proposed approach, along with the calculation accuracy with respect to the true values. For instance, a dot with a radius of 10 μm has a true area of 314.159 $\mu m^{2}$, and the estimated area of 313.2576 $\mu m^{2}$ yields an accuracy rate of 99.7%. The proposed algorithm yielded similar area estimation accuracy for all dot sizes.

The distance of the neighboring dots along the counterclockwise printing direction was quantified for analyzing dot spacing. The overall distance quantification accuracy was 94.7 ± 1.2%. For example, Table 4 shows the distance information of the dots labeled in Figure 5. As can be seen in Table 4, the distance quantification also achieved high accuracy.

## 5. Discussion

**Figure 6:**
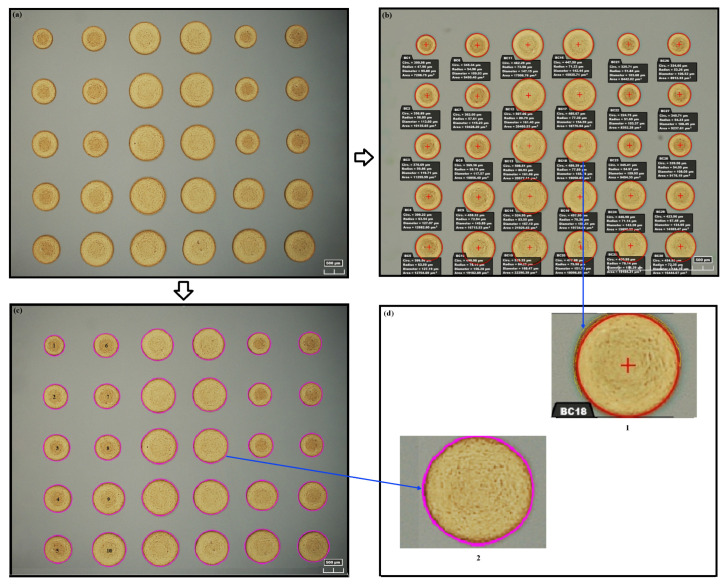
Experimental EHD DoD print result analysis. (**a**) The origin image of the printed droplet. (**b**) The printed droplet was analyzed offline using a manual microscope approach. (**c**) The analyzed image using the the proposed approach. (**d**) Detailed comparison of the two analysis methods.

After the accuracy of the proposed algorithm was validated, we demonstrated the efficacy of this approach in analyzing experimental EHD DoD printed results and compared it with the conventional manual microscope analysis method. The EHD DoD printed dots are shown in Figure 6.

Traditionally, microscope analysis of EHD DoD printing is accomplished by manually outlining the dot contours using default circular shapes, allowing the radius and area calculations as shown in Figure 6b. For the same image of printed dots, the proposed analysis approach found the exact shape of the dots automatically using feature extraction and edge detection methods to extract and trace boundaries, as shown in Figure 6c. During the edge detection step, the image was processed to highlight the edges between the dots and the background. Following edge detection, the borders of the dots were detected and tracked. The contour points of the edges were then sorted into a vertex list containing the boundary coordinates. The dot areas were then calculated. Meanwhile, a contour shape was drawn to visualize the detected boundaries surrounding all connecting curves. It is clear that the proposed approach is based on the true shape of the printed dots rather than assuming circular shapes as in the conventional manual microscope method.

A detailed comparison of the two methods is shown in Figure 6 and Table 5. For example, ten dots were selected from the EHD DoD printing results image. The size calculation comparison of these two methods is shown in Table 5. The manual microscope results were generated by carefully outlining each dot from the experimental list and then computing the area of the outline circles. Using the manual microscope result as a reference, the dot areas quantified by the proposed approach are very close (see the last column of Table 5). The manual approach could achieve accuracy because the experimental list drew the circular outline to match the dot edge as closely as possible. However, there are cases where the precision cannot be guaranteed.

As highlighted in Figure 6d, the yellow contour shows the undetected portion of the dot on which the red circle was the manual detection result. This is because the accuracy of this approach entirely relies on human eye inspection, and the detection is only restricted to round circles. Therefore, the manual process may lead to an inaccurate estimation of the dot shape and size. Moreover, such a detection usually takes at least several seconds for each dot, thereby at least **several minutes** for the entire image. On the other hand, the proposed approach detected the dot based on its actual shape with no pre-defined profile, and the detected contour precisely matched the edge of the dot. The close match of the outline with the printed dot edge across all samples indicates high precision and reliability. Analyzing the entire image only took a **few milliseconds**.

Therefore, the proposed approach outperforms the conventional manual microscope analysis approach in terms of both accuracy and efficiency. To further improve the capability of the proposed method, we will optimize the algorithm by exploring more shape detection options, such as RANSAN [45], J-Linkage [46], and CNNs [47], to further improve its quantification efficiency. It is noted that the proposed method is not limited to EHD DoD printing results analysis. It can be easily adapted for any applications that involve isolated pattern detection and size quantification.

Although the proposed method is demonstrated to have high accuracy under controlled conditions, several limitations may impact its effectiveness in broader applications. One major limitation is the dependence on high-quality imaging. Changes in lighting conditions, focus inconsistencies, or picture resolution limits can affect the edge and contour detection processes, potentially reducing accuracy. External conditions, for example, temperature or humidity of the environment, could also further influence the formation of the ink droplets, resulting in the shape of droplets that could make it difficult for an algorithm to trace boundaries and centroid identification accurately.

## 6. Conclusions

This paper presented an algorithm that uses feature extraction techniques to address the printing result analysis challenges of EHD DoD manufacturing. The proposed technique, which employs edge and contour detection methods, facilitates significant improvements in detecting printed dot boundaries, resulting in high accuracies in dot size and spacing quantification. The proposed approach was first validated using simulated EHD DoD dot arrays. The achieved high accuracy in dot area and distance quantification demonstrated the reliability of our approach. Comparison with the conventional manual microscope-based method on EHD DoD-printed images further demonstrated the efficacy of the proposed approach.

For future applications, the proposed method can be integrated into real-time EHD printing systems to improve printing quality analysis. For example, the dot quantification results obtained from the proposed algorithm can be used as the feedback data for closed-loop EHD printing system control. The difference between the desired dot specifications and the quantified results will be used by the controller to adjust the printing parameters (such as jetting frequency/voltage, printing nozzle-substrate distance, and printing stage moving speed) to optimize the printing performance in real time. Potential challenges involve computational speed optimization for achieving real-time processing and accurate modeling of the printing parameters vs. dot specification relation. Future work will focus on seeking both software and hardware approaches to address these challenges toward real-time EHD DoD printing optimization.

## Figures and Tables

**Table 1:** Dot area calculation accuracy for simulated EHD DoD line pattern printing.

Dot	Radius (μm)	True Area ($\mu m^{2}$)	Estimated Dot Area ($\mu m^{2}$)	Accuracy %
1	30	2827.4	2880.1	98.1
2	35	3848.4	3846.1	99.9
3	40	5026.5	4952.7	98.5
4	45	6361.7	6159.2	96.7
5	50	7853.9	7491.6	95.1
6	25	1963.5	2081.1	94.3
7	20	1256.6	1396.4	89.9
8	15	706.8	837.8	94.3
9	55	9503.3	8959.5	93.9
10	60	11,309.7	10,581.7	93.2

**Table 2:** Dot distance calculation accuracy and categorization for simulated EHD DoD line pattern printing.

Distance from Center to Center	True Distance (μm)	Estimated Distance (μm)	Distance Profile	Overall Accuracy %
1–2	83.9	82.1	Close	97.8
2–3	97.0	96.1	Close	98.9
3–4	100.3	99.2	Close	98.8
4–5	120.1	117.8	Satisfactory	98.0
5–6	123.3	120.9	Satisfactory	97.9
6–7	90.4	89.9	Close	99.3
7–8	80.6	79.0	Close	98.0
8–9	118.4	116.2	Satisfactory	98.1
9–10	156.2	153.4	Satisfactory	98.1
11–12	136.7	134.8	Satisfactory	98.5
Average				98.3 ± 0.4

**Table 3:** Dot size calculation accuracy for simulated EHD DoD circular pattern printing.

Dot	Radius (μm)	True Area ($\mu m^{2}$)	Estimated Dot Area ($\mu m^{2}$)	Accuracy %
1	12	452.389	446.114	98.6
2	11	380.1	375.9	98.9
3	10.5	346.3	342.7	98.9
4	10	314.1	313.2	99.7
5	9.5	283.5	282.7	99.7
6	9	254.4	255.9	99.4
7	8.5	226.9	227.7	99.6
8	8	201.0	202.1	99.4
9	7.5	176.7	178.4	99.0
10	7	153.9	155.9	98.6
11	6.5	132.7	136.2	97.3
12	6	113.1	115.8	97.5

**Table 4:** Dot distance calculation accuracy for simulated EHD DoD circular pattern printing.

Distance from Center to Center	Location (μm)	True Distance (μm)	Estimated Distance (μm)	Overall Accuracy %
1–2	(364, 438)	37.06	39.02	94.7
2–3	(436, 671)	30.53	32.03	95.0
3–4	(625, 737)	28.10	29.53	94.9
4–5	(807, 768)	28.39	29.66	95.5
5–6	(992, 756)	34.31	36.09	94.8
6–7	(1201, 671)	39.05	40.86	95.3
7–8	(1291, 432)	38.87	40.73	95.2
8–9	(1184, 201)	25.90	27.19	95.0
9–10	(1033, 123)	29.00	30.29	95.5
10–11	(844, 112)	34.05	35.74	95.0
11–12	(621, 125)	30.15	31.63	95.0
12–1	(446, 217)	41.64	37.71	90.5
Average				94.7 ± 1.2

**Table 5:** Size Detection of EHD Printed dots.

Dot	Microscope Radius (μm)	Microscope Area ($\mu m^{2}$)	Area from Proposed Algorithm ($\mu m^{2}$)	Difference in Percentage %
1	47.90	7208.75	6885.90	95.3
2	56.80	10,135.65	10,422.83	97.1
3	59.86	11,255.59	11,800.95	95.1
4	63.54	12,682.60	12,537.01	98.8
5	63.59	12,704.69	12,875.63	98.6
6	54.96	9490.40	10,051.13	94.4
7	57.61	10,428.20	10,419.68	99.9
8	58.97	10,856.40	10,790.33	99.3
9	72.94	16,715.53	17,123.40	97.5
10	78.14	19,182.89	18,756.68	97.7

## Data Availability

The original contributions presented in the study are included in the article; further inquiries can be directed to the corresponding author.

## Abbreviations

The following abbreviations are used in this manuscript:

EHD	Electrohydrodynamic
DoD	Drop-on-Demand
AM	Additive Manufacturing
GUI	Graphical User Interface
OpenCV	Open Source Computer Vision Library
SolidWorks	Solid Modeling Computer-aided Design and Computer-aided Engineering Program
